# Comparative Assessment of Tungsten Toxicity in the Absence or Presence of Other Metals

**DOI:** 10.3390/toxics6040066

**Published:** 2018-11-09

**Authors:** Ola Wasel, Jennifer L. Freeman

**Affiliations:** School of Health Sciences, Purdue University, 550 Stadium Mall Drive, West Lafayette, IN 47907, USA; owasel@purdue.edu

**Keywords:** tungsten, cobalt, metal mixtures, nickel, toxicity

## Abstract

Tungsten is a refractory metal that is used in a wide range of applications. It was initially perceived that tungsten was immobile in the environment, supporting tungsten as an alternative for lead and uranium in munition and military applications. Recent studies report movement and detection of tungsten in soil and potable water sources, increasing the risk of human exposure. In addition, experimental research studies observed adverse health effects associated with exposure to tungsten alloys, raising concerns on tungsten toxicity with questions surrounding the safety of exposure to tungsten alone or in mixtures with other metals. Tungsten is commonly used as an alloy with nickel and cobalt in many applications to adjust hardness and thermal and electrical conductivity. This review addresses the current state of knowledge in regard to the mechanisms of toxicity of tungsten in the absence or presence of other metals with a specific focus on mixtures containing nickel and cobalt, the most common components of tungsten alloy.

## 1. Introduction

The perceived stability of tungsten (W) in the environment led to its use as a “green bullet”; however, soon after replacing lead in ammunitions, W began to be detected in soil and ground water near military sites, indicating W’s mobility in soil. The solubility of W in soil depends on the pH. Alkaline soil leads to an increase in the solubility of W, which increases the probability of contaminating groundwater [1,2]. Other factors can affect solubility of W in soil, such as the type of minerals present [3]. Various trends of W adsorption were observed depending on the type of the clay mineral and pH. Compared to kaolinite and montmorillonite, illite had the lowest adsorption capacity to monotungstate [3]. Therefore, pH and type of clay minerals control the solubility of W in the soil, which eventually can move into drinking water supplies.

There is currently no limit set for W in drinking water in the United States or by the World Health Organization [1]. There are only regulatory limits for occupational W exposure that are provided by the American Conference of Governmental Industrial Hygienists (ACGIH), the National Institute of Occupational Safety and Health (NIOSH), and the Occupational Safety and Health Administration (OSHA). The United States Environmental Protection Agency (U.S. EPA) did claim W as an emerging toxicant as a consequence of studies that reported adverse health outcomes associated with environmental W exposure. 

The potential association between W environmental contamination and a childhood cancer cluster in Churchill County, Nevada, brought W toxicity into question and into the spotlight in the period between 1997 and 2002. The level of W in urine in the population of this county and the concentration of W in the drinking water (0.25–337 µg/L) was reported to be higher than in other areas [4]. Churchill County is located near W mines and military bases, which led to confusion on whether the cause of elevated W was of natural or anthropogenic sources. Based on the initial reports, the U.S. Centers for Disease Control and Prevention (CDC) completed a study to examine three other communities in Nevada: Lovelock, Pahrump, and Yerington. These towns were chosen based on their similarity to Churchill County in mining activity and hydrogeological properties. During the study, water, dust, and soil from the yard of homes were collected from selected households and level of W measured. Urine samples were also collected from community members. The results showed that the level of W in these three counties was elevated according to the National Health and Nutrition Examination Survey (NHANES) reference population (0.48 µg/L). Also, the results revealed that people of Churchill County may be exposed to higher levels of W due to the fact that Nevada is naturally rich in W [4]. Sheppard et al. [5] explained through tree ring analysis that the elevation of W and cobalt (Co) in trees was coincidental with the onset of the leukemia cases in Fallon County. On the other hand, another article pointed to the fact that tree ring W concentrations in Fallon cottonwoods were between 40,000 and 70,000 µg/L between 1989 and 2000 and an elevated concentration (180,000 µg/L) was observed over the period 2001–2004, indicating that the onset of leukemia cases occurred before the increase in W levels. These conflicting results raised additional questions regarding the toxicity of W. Furthermore, a study showed that implanted weapon grade W alloy, containing 91.1% W, 6% Ni and 2.9% Co, in rats caused formation of tumors around the implant in 100% of the treated rats after one month of implantation; however, the 100% Ni implant caused formation of tumors at a slower rate [2,6]. This study coupled to the finding in Fallon County, Nevada led to heightened concerns surrounding the toxicity of W in the absence or presence of other metals. 

## 2. Tungsten: Uses and Routes of Exposure

Tungsten exposure can occur through natural or anthropogenic sources. W is widely used in a variety of applications based on the unique properties of this metal. W is considered as a refractory metal since it has the highest melting temperature of all metals. W also has the lowest vapor pressure, the highest tensile strength at high temperature (over 1655 °C), and the lowest coefficient of expansion compared to all other metals [7]. W is resistant to corrosion and mineral acids can affect W only slightly at high temperatures [7]. W is also a good electric conductor. Based on these unique characteristics, W is used in alloy manufacturing, high speed tools, light filaments, X-ray shielding, welding electrodes, solar energy devices, and pigment production. The most common form of W is tungsten carbide, which is used in making cutting tools [1]. 

Exposure to W can be from natural or anthropogenic sources. Occupational exposure occurs via dust inhalation during mining W metal from the ore and during preparation of tungsten carbide products. The general population can be exposed through inhalation of W dust in the air or drinking contaminated water. Water contamination comes from dissolved rocks or through effluent of mining sites and hard metal industries’ waste, but since W is not regulated in drinking water, W data in water are limited [1]. Exposure to W in waste sites through contaminated soil is also reported [1]. It is known that W ions are not metabolized in the body and that 90% of inhaled W is eliminated in urine after 14 h of exposure [8]. W in blood levels has been measured to be 1–6 µg/L and the urine level is 0.085 µg/L in the general population. [1]. Also, W can cross the placenta and transport from mother to fetus [1]. 

Occupational exposure to W during mining processes or hard metal industries is one the most important routes of exposure via inhalation of W dust [1]. Higher W concentrations were detected in lungs, blood, and urine of individuals who worked in hard metal plants as compared to individuals who worked in other industries [9]. A study examining workers who were exposed to W during different tasks of the hard metal industry, showed that the mean concentration of W in the lung is 107 ng/g, 1.35 ng/g in blood, and 12 ng/g in urine. The levels in control groups were 1.5 ng/g in the lung, 0.4 ng/g in blood, and 0.7 ng/g in urine [9]. The concentration of W in the air of 15 welding shops in Montréal, Quebec was between 0.15 and 1.50 μg/m^3^, whereas the urban air W concentration was 0.0052 μg/m^3^ [10]. 

## 3. Tungsten Toxicity

Since one of the primary routes of occupational exposure to W is inhalation, many studies have used lung cells to assess W toxicity. A study by Laulicht et al. [11] used immortalized human bronchial epithelial cells (Beas-2B) and assessed ability of these cells to grow independently on soft agar to migrate and to induce tumor formation in vivo in the absence and presence of W. Beas-2B cells were treated with 0, 50, or 250 µM sodium tungstate (Na_2_WO_4_) and after six weeks of treatment, the cells were incubated in soft agar without Na_2_WO_4_. The results revealed that the number of colonies resulting from control cells (0 µM Na_2_WO_4_) was significantly lower than treated cells, indicating the ability of W-treated cells for anchorage-independent growth, an indicator of tumorigenicity of cells [11]. Tungstate-treated cells were also able to heal a wound 20 h after a scratch, indicating the ability of the cells to migrate [11]. The authors of this study then injected Na_2_WO_4_ transformed cells into flanks of six-week-old female athymic nude mice. After one month of injection, 100% of tungstate-transformed cells resulted in visible tumors [11]. On the other hand, control cells did not induce any tumor. RNA-sequencing of isolated colonies revealed that many genes associated with respiratory tract cancer were altered by tungstate treatment, including upregulation of NADPH dehydrogenase quinone 1 (*NQO1*). This result was consistent with other studies that showed the dysregulation of *NQO1* is associated with lung cancer [12,13]. S-phase kinase-associated protein 2 (*SKP2*), a gene in which increased expression enhances the growth of small cell lung cancers, was also upregulated [14]. Furthermore, cyclin-dependent kinase inhibitor 1A (*CDK1A*), which encodes the cell cycle regulator p21, was downregulated. Repression of *CDK1A* leads to many types of cancers due to inhibition of caspase activity and apoptosis. Overall, the results of this study showed the carcinogenic potential of W. In a follow-up study, the authors found that W exposure increased histone methylation of H3K4me3 and H3K9me2 by causing loss of the histone demethylase dioxygenases, Jumonji-AT rich interactive domain 1A (JARID1A) and Jumonji domain containing 1A (JMJD1A), in BeasB1 cells [15]. The authors explained that the observed increase in methylated histones was not due to an increase in methylation, because with a depletion of S-adenosyl-methionine (SAM), the coenzyme for histone methyltransferase, an increase in methylated histones was still observed [15]. In addition, rats exposed to Na_2_WO_4_ in drinking water showed an increase in H3K4me3 and H3K9me2 in livers. The authors suggested that inhibition of demethylases may be due to proteasome activity leading to degradation of these enzymes, or by induction of reactive oxygen species (ROS) [15]. 

**Figure 1:**
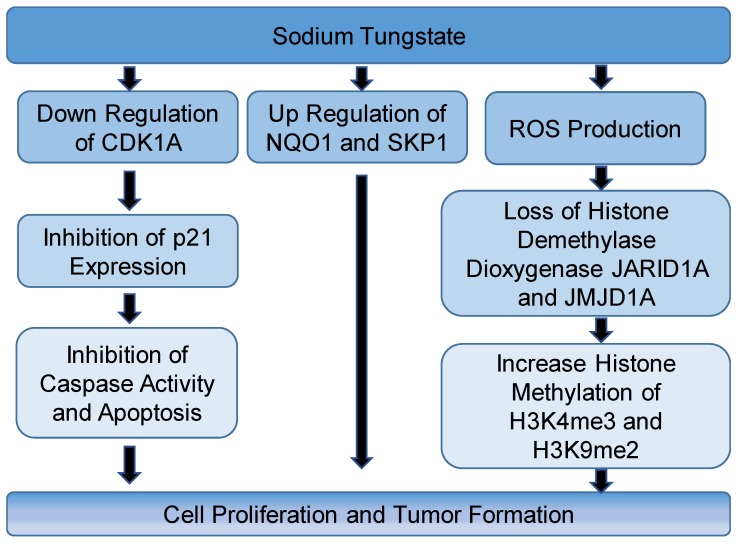
Summary of the proposed mechanisms of sodium tungstate toxicity. CDK1A: cyclin-dependent kinase inhibitor 1A; JARID1A: Jumonji-AT rich interactive domain 1A; JMJD1A: Jumonji domain containing 1A; NQO1: NADPH dehydrogenase quinone 1; ROS: reactive oxygen species; SKP: S-phase kinase-associated protein.

W was detected in patients’ urine who had a W-based shield in their breasts (including patients with a mastectomy), indicating the mobilization of W into other organs [16]. These results were confirmed in a 66Cl4 cell model and in vivo using female BALB/c mice that were administered 15 ppm Na_2_WO_4_ in drinking water [16]. W did not enhance proliferation of 66CL4 cells, but led to lung metastasis following injection of 66Cl4 cells into the fourth mammary fat pad [16]. The results found that W induced changes that stimulate metastasis such as activated fibroblasts, matrix metalloproteinases, and myeloid-derived suppressor cells. The authors suggested that Na_2_WO_4_ induced breast cancer metastasis by changing the microenvironment of the tumor. Taking all of these results together, W has a carcinogenic potential that should be taken into consideration for regulatory standards. Figure 1 summarizes the possible mechanism of toxicity of sodium tungstate. 

Furthermore, a study of tungsten oxide (WO_3_) nanoparticles showed that only an oral dose of 1000 mg/kg of WO_3_ 53.2 nm particles to Wistar rats induced DNA damage, micronuclei formation, and reduction of glutathione and catalase [17]. The authors explained that only a high dosage of WO_3_ nanoparticles induced the toxic effects, signifying differential toxicity based on particle size, which should also be considered. 

## 4. Toxicity of Tungsten Metal Mixtures

W is usually used in the form of tungsten carbide (WC)-Co or as an alloy with other metals. WC is formed by binding W to one carbon atom. WC is used in many applications such as cutting tools and mining equipment due to its hardness [18]. W is added to other metals to increase their hardness and improve their properties. Research studies support increased toxicity of W when present in a metal mixture (Table 1). Signs of memory and sensory deficits was reported among workers in the hard metal industry who were exposed to hard metal dusts consisting of (79–95%) WC, 10% Co, and small amounts of other metals such as vanadium, chromium, and/ or titanium [19,20,21]. Previously, it was perceived that Co was the main cause of the adverse health outcomes in hard metal workers [22,23], although tungsten oxide was detected in the air of hard metal workplaces, indicating a potential role in the lung and neurological effects [24,25]. 

### 4.1. Tungsten and Cobalt Mixtures

Cobalt (Co) is a natural trace element that is present between nickel (Ni) and iron (Fe) in the periodic table and shares some of their properties. Co is considered as an essential element since the body cannot synthesize this element. Co can be used as a treatment of anemia using 0.16–1 mg Co/kg [1]. It was reported that Co biomagnifies through the food chain since this element can accumulate in fruits and grains. People can be exposed to Co by inhalation of Co dust, or through ingestion of food or drinking water contaminated with Co. High level of exposure mainly occurs near mining, smelting facilities, and hazardous waste sites [1]. The concentration of Co in soil ranges from 1 to 40 ppm. According to the Agency of Toxics and Disease Registry (ATSDR), the concentration of Co in drinking water is 1–2 parts per billion (ppb) and may reach 100–1000 ppb in contaminated areas [1]. After Co enters the body, some is excreted through feces, but the remaining part is distributed into all tissues and mainly accumulates in bones, liver, and kidney [1]. Use of metal-on-metal implants raised big concerns due to chronic exposure to Co. Animal studies showed that implanted Co particles caused formation of tumors. According to the International Agency for Research on Cancer (IARC), Co is considered as possibly carcinogenic to humans (group B2), but Co with WC is considered as probably carcinogenic to humans (group A2) [26].

Lombaert et al. [27] evaluated the apoptogenic potential of Co particles, WC, WC-Co particles, and cobalt chloride (CoCl_2_). The authors exposed human peripheral blood mononucleated cells (PBMCs) to 2.0–6.0 µg/mL of Co alone or as WC-Co and 33.3–200 µg/mL WC alone. In their study, an early apoptotic effect was determined by Annexin V staining after 15 min and 6 h post exposure [27]. Results revealed that the four forms of metals all induced apoptosis. Interestingly, it has been found that the relative apoptotic activity of CoCl_2_ and metallic Co are almost the same. It was reported that 77% of Co solubilized from metallic Co into the cell-free media after 15 min of exposure and complete solubilization was observed after 24 h [27]. It was also shown that WC-Co has differential sensitivity to apoptosis inhibitors [27]. The percentage of apoptosis inhibition significantly increased after adding caspase 9-inhibitor, but not caspase 8 to cells treated with WC particles [27]. On the other hand, the percentage of apoptosis inhibition significantly increased after adding both caspase 8 and caspase 9-inhibitor to cells treated with Co [27]. This differential sensitivity to caspase inhibitors suggested different mechanisms by which WC particles and Co induce apoptosis. Lombaert et al. observed that WC was internalized by mononuclear cells and was not solubilized in the media, suggesting phagocytosis-mediated apoptosis [27]. Caspase 8 is involved in the extrinsic apoptotic pathways via death receptors and then induces intrinsic pathways [28]. As concluded by Lombaert et al. the significant increases in apoptotic DNA fragmentation after exposure to WC-Co for 24 h compared to Co, CoCl_2_, or WC alone can be attributed to the combined effect of WC and Co [27]. However, relative apoptosis frequency using Annexin V staining after 6 h of exposure indicated similar or lower effect of WC-Co compared to Co and WC, respectively [27]. In 2008, the same group showed that WC-Co and Co particles had the same responses in upregulating apoptosis and stress responses and downregulation of immune responses pathways after 24 h of exposure in human peripheral blood mononuclear cells (PBMC) [29]. The authors suggested that the long period of exposure (24 h) led to complete solubilization of Co from WC-Co particles, which may lead to masking of the effect of the WC-Co particle itself and showed the effect of Co ions. Based on these previous results, the authors decided to use a short period of exposure (15 min) as a model of exposure to understand the effect WC-Co particles [30]. Human PBMCs were exposed to 6 µg/mL equivalent Co of WC-Co, CoCl_2_ or Co particles for 15 min. The results revealed that a cascade of events, including p38/mitogen-activated protein kinase (MAPK) activation, hypoxia inducible factor 1-α (HIF-1α) stabilization, heme oxygenase 1 (HMOX1) transcriptional activation, and p53 stabilization occurred upon exposure to WC-Co or Co particles only, but not WC alone or CoCl_2_ [30]. These results indicated that the toxicity of WC-Co was due to the production of ROS. Other studies showed that stabilization of p53 occurred as a response to ROS production [31,32]. The effects of Co particles were slower compared to WC-Co particles, thus confirming that WC and Co interaction caused rapid release of ROS. The same study showed that CoCl_2_ could not induce HIF-1α stabilization, but WC-Co and Co particles did induce HIF-1α stabilization [30]. This can be explained by ROS that induce induction of phosphatidyl inositol 3 kinase (PI3K), or protein kinase B (Akt) pathways which induce HIF-1α stabilization [33,34,35]. It was reported that Co can be involved in redox cycling reactions and ROS can be generated by Fenton/Haber–Weiss chemistry [36]. On the other hand, Lison et al. [37] proposed a mechanism for the interaction of Co and WC particles in which W particles reduced oxygen using electrons from Co and then produced ROS. They suggested that Co ions are a product of the interaction but did not directly cause the production of ROS [37]. This mechanism explains why Co ions or tungstate ions did not affect the viability of different types of human cells and only WC-Co nanoparticles (10% weight content Co) caused cytotoxic effects [38]. Another possible mechanism that explains the higher toxic effect of WC-Co nanoparticles compared to either WC or Co alone is that nanoparticles act as a carrier, which helps ions to penetrate the cell membrane [39]. This mechanism could explain the increase in Co uptake from WC-Co compared to Co metal alone [40]. One can conclude that production of ROS may be due to the synergistic effect of Co ions and the interaction of Co and W surfaces. 

**Figure 2:**
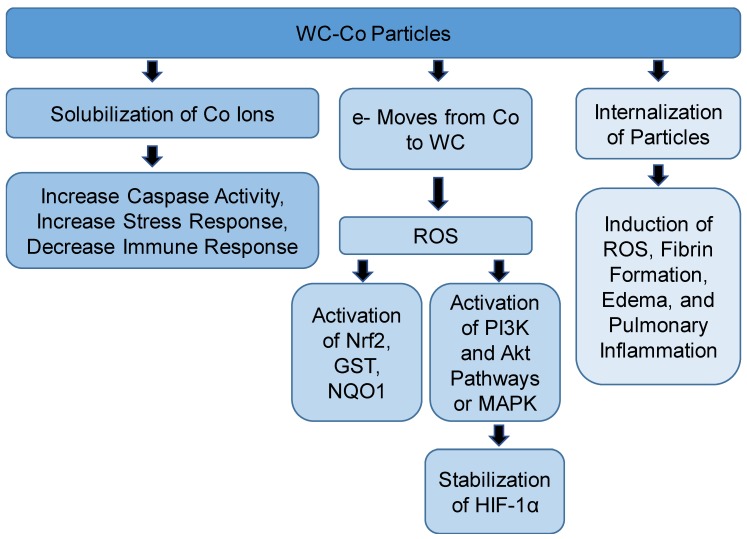
Summary of the proposed mechanisms of tungsten carbide-cobalt (WC-Co) toxicity. Akt: protein kinase B; GST: glutathione S-transferase; HIF-1α: hypoxia inducible factor 1-α; MAPK: mitogen-activated protein kinase; NQO1: NADPH dehydrogenase quinone 1; Nrf2: nuclear factor erythroid 2-like factor 2; PI3K: phosphatidyl inositol 3 kinase; ROS: reactive oxygen species.

However, Busch and coworkers found that gene expression cannot explain the differential toxicity of WC-Co nanoparticles and CoCl_2_ [41]. Their study demonstrated that immortalized human keratinocytes (HaCaT) treated with WC-Co nanoparticles or CoCl_2_ showed increased transcription of genes involved in the hypoxia pathways compared to WC, emphasizing the role of Co ions in this response [41]. Zhang et al. showed that WC-Co nanoparticles activated nuclear factor erythroid 2-like factor 2 (*Nrf2*) and its downstream genes such as glutathione S-transferase (*GST*) and *NQO1* after 24 and 48 h of exposure in mouse epidermal JB6 cells [42]. After treating the cells with catalase, inhibition of expression of these genes was observed. This result indicates that ROS was responsible for the activation of *Nrf2*, *GST*, and *NQO1* [42]. Nrf2 is a transcription factor that regulates the expression of antioxidants and detoxification enzymes [43]. Depletion in glutathione was observed by WC-Co, indicating generation of oxidative stress [44]. An interesting study showed that WC-Co nanoparticles could be used as a positive control of induction of genotoxicity [29] (Figure 2). 

The size of WC-Co particles plays an important role in the observed effects of exposure as well as the mechanism of toxicity. In a study using Beas-2B cells to assess the toxicity of nano or micro WC-Co particles, cells were treated with 0, 10, 100, or 1000 µg/mL for 0, 0.5, 1, 2, 6, 12, or 48 h, and cell viability using the MTT assay (a colorimetric assay to determine metabolic activity), Annexin-V apoptosis, and oxidative stress was determined [45]. The results revealed that exposure to WC-Co particles led to a decrease in the viability of the cells compared to control. Also, it was observed that the cell viability for nanoparticle-treated cells was lower than cells treated with microparticles at all exposure periods except at 48 h. This result can be explained by increasing the number and surface area of nanoparticles, which increases the toxicity of the particles. Also, the oxidative stress increased as the concentration of the particles increased. A significant increase in oxidative stress was observed when cells were exposed to 1000 µg/mL of WC-Co particles. Dose-dependent induced apoptosis was observed after exposure to WC-Co particles, but a significant increase compared to control was only observed after exposure to 1000 µg/mL. This study suggested that apoptosis induced by exposure to WC-Co particles may contribute to hard metal lung disease (HMLD) progression and lung cancer. The study also indicated that a possible mechanism of toxicity of nano WC-Co particles is the internalization of the particles, which may explain the hard metal deposits present in lungs in HMLD. A-Rahman et al. found that intratracheal instillation of hard metal that contains Co, chromium, and Fe leads to edema, fibrin formation, and induction of pulmonary inflammation and increased nitric oxide production via changes in nitric oxide synthase pathways [46]. Figure 2 shows the mechanisms of toxicity of WC-Co particles. 

The size of WC-Co particles was examined by Ding et al. [47]. They treated JB6P+ cells with nano (80-nm) and fine-sized (4-µm) particles. The results showed an increase in activation of transcription factors including activator protein (Ap-1) and nuclear factor kappa-light-chain-enhancer of activated B cells (Nf-κB), MAPK signaling pathways, and a decrease in glutathione (GSH) levels with nano-sized WC-Co treatment compared to fine size, suggesting that higher oxidative stress was produced with exposure to nano WC-Co particles [47]. These results are consistent with those of Zhao and coworkers who found that WC-Co nanoparticles stimulated more ROS production and induced extrinsic and intrinsic apoptotic pathways compared to fine particles in rat JB6 cells [48]. Even within the nanoscale, differential modes of action were seen. It was observed that WC particles (mean size = 113 ± 2 nm) induced the formation of ROS and micronuclei, indicating chromosomal instability, in HepG2 and HaCat cells; however WC or WC-Co particles (mean size = 145 ± 5 nm) did not induce ROS generation or genotoxicity [49]. The authors explained the increase in ROS production was due to the increased surface area of smaller WC particles. This study concluded that the toxicity of the particle does not depend on presence or absence of Co, but mainly on the size of the particles [49]. The different processes of production may affect the toxicity of WC particles. The latter study pointed to the presence of carbon black in the WC nanoparticles [49]. Carbon black is a possible carcinogen to humans based on IARC classification and may contribute to the genotoxicity potential of WC particles [26]. These results highlight the importance of taking production processes into consideration, while studying the toxicity of WC nanoparticles as well as other factors such as size and composition. 

Despite all of these previous studies that show the potential toxic effects of WC-Co particles, a study showed that nano-WC-Co coating may lead to a better environmental impact compared to chromium coating [50]. This study used life cycle analysis to compare WC-Co coating and chromium coating. The authors not only focused on the toxicity during production, but emphasized the overall effects of WC-Co in the environment, such as energy demand, metal depletion, and freshwater eutrophication.

### 4.2. Tungsten, Cobalt and Nickel Mixtures

Studies showed that W/Ni/Co alloy has different toxicity compared to Ni or Co alone and a specific ratio of the three metals cause significant effects (Table 1). Two doses (4 pellets and 20 pellets) of weapon-grade W alloy (WA) (91.1% W, 6% Ni, and 2.9% Co) or 100% Ni pellets were implanted intramuscularly into F344 rats to mimic the effect of shrapnel wound [5]. Results revealed an increase in red blood cells (RBCs), white blood cells (WBCs), neutrophils, lymphocytes, and monocytes after 1 month and tumor formation in 100% of rats after 4–5 months post-implantation of the high dose of WA. The low dose WA and Ni pellet induced tumor, but at a lower rate. The tumors were classified as high grade pleomorphic rhabdomyosarcomas. Interestingly, the authors concluded that this effect was not due to Co or Ni because 100% Ni alloy induced tumor formation after a longer time compared to WA that contains only 6% of its components as Ni. These results suggested a synergistic effect of the three metals to form free radicals at the interface of pellets and tissue [6]. Also, another study showed that Co can induce polycythemia in rats, but at a higher concentration compared to Co concentration in W alloy [60]. These results suggest a synergistic effect of the three metals is the cause of the observed effects.

As a trial to understand the mechanism of toxicity of WA, a comparison was performed using different types of W/Ni/Co alloys and individual metals. The results showed that treatment of rat skeletal muscle cells line (L6-c11) with W/Ni/Co (91% W: 6% Ni: 3% Co), W/Ni/Co (97% W: 2% Ni: 1% Co), or W/Ni/Fe (97% W: 2% Ni: 1% Fe) alloys caused inhibition of activity of caspase 3 and induced production of ROS and DNA damage [52]. Only W/Ni/Co (91% W: 6% Ni: 3% Co) caused significant DNA damage compared to control [52]. A treatment using Co only induced a lower level of ROS generation compared to WA. The researchers of this study suggested that in addition to the production of ROS, dissolution of Co^2+^ ions and Ni^2+^ ions may lead to stabilization of HIF proteins that can induce regulation of gene expression; such as the induction of vascular endothelial growth factor (VEGF) and inhibition of caspase 3 [52]. An earlier study in which 1HAEo cells were treated with Co^2+^or Ni^2+^ showed a decrease in intracellular ascorbate post exposure. The results also showed that addition of ascorbate with Co increased intracellular ascorbate, which resulted in reversing stabilization of HIF-1α and HIF-1-dependent gene transcription [61]. Ascorbic acid is essential for HIF-1α activity. Ascorbic acid is a cofactor of the prolyl hydroxylases that is required to hydroxylate proline residues on oxygen-dependent degradation domain on HIF-1α [61]. The authors explained that the presence of ascorbate in prolyl hydroxylases keeps iron in Fe^2+^ form, which is important for active enzymes [61]. Exposure to metals such as Co^2+^or Ni^2+^ leads to the oxidation of ascorbate into dehydroascorbic acid and production of ROS. In the case of ascorbate depletion, Fe^2+^ is converted into Fe^3+^ and inactivation of prolyl hydroxylase and inhibition of HIF-1α degradation occurred [61]. 

Schuster and coworkers showed that W/Ni/Fe (97.1% W: 1.7% Ni: 1.2% Fe) alloy did not induce tumor formation around implanted alloy pellets in F344 rats [53]. They observed a high increase in metal concentrations in urine in the case of the W/Ni/Co (91.1% W: 6% Ni: 2.9% Co) alloy, but not in the W/Ni/Fe alloy, which dissolved more slowly and produced fewer ROS compared to W/Ni/Co alloy, indicating that the mobilized metals were the causal factor in tumor formation [53]. Therefore, the authors of this study suggested that designing corrosion-resistant alloy may solve the concerns around the long-term effects. A similar result was observed using a human skeletal muscle cell (hSkMC) line, where no oxidative stress-related transcriptional changes occurred with W/Ni/Fe treatment [54]. It was explained that the low toxic effects of W/Ni/Fe alloy may be due not only to the absence of Co but also, the low concentration of Ni [53]. This can be confirmed by the fact that W/Ni/Co (97% W: 2% Ni: 1% Co) alloy has lower toxicity compared to W/Ni/Co (91% W: 6% Ni: 3% Co) in human and rat skeletal muscle cells [54]. When hSKMCs were treated with W/Ni/Co (91% W: 6% Ni: 3% Co), inhibition of muscle-specific protein transcription was observed, in addition to induction of hypoxia, stress response, glycolysis, and angiogenesis-related genes. All of these observations are consistent with the expected outcomes of hypoxia-induced by Co. It is noteworthy that WA (91% W: 6% Ni: 3% Co) induced a more toxic effect compared to WA (97% W: 2% Ni: 1% Co) [52]. This result highlights that the composition of the alloy could affect its toxicity and by changing composition, toxic effects could be avoided. Another study showed a different result for W/Ni/Fe, when Roedel et al. studied the effect of intratracheal instillation of the W/Ni/Co alloy (91% W: 6% Ni: 2.9% Co), or the W/Ni/Fe alloy (92% W: 5% Ni: 3% Fe) and each individual metal alone in rats [55]. Phagocytosis of metal particles by lung macrophages and influx of neutrophils were observed in both W/Ni/Co alloy and W/Ni/Fe alloy treatment groups, but not in the W alone treatment. The results also showed that the induction of the production of inflammatory cytokines and generation of ROS 24 h post instillation of W/Ni/Co alloy and W/Ni/Fe alloy [55]. The difference in Ni concentration in W/Fe alloy in other studies may explain the different results. Also, the different cell types may have a different response. Studying the effect of WC-Co nanoparticles on different human cell lines showed that astrocytes are the most sensitive cells to the cytotoxic effects, indicating that the nervous system could be the most affected body system upon exposure to these particles, [38]. Another study showed that exposure to the W/Ni/Co alloy induced epigenetic alterations in hippocampal primary neuronal cultures [59]. The authors suggested that the three metals are interacting synergistically because the observed H3-Ser10 dephosphorylating due to W/Ni/Co alloy was significantly compared to Co- alone-treated cells [59].

**Figure 3:**
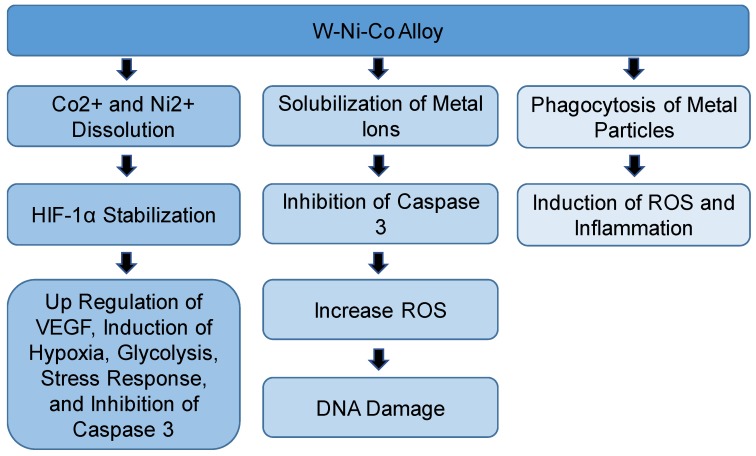
Summary of the proposed mechanisms and effects of the tungsten-nickel-cobalt (W-Ni-Co) alloy. HIF-1α: hypoxia inducible factor 1-α; ROS: reactive oxygen species; VEGF: vascular endothelial growth factor.

It was reported that induction of inflammatory cytokines and macrophages may lead to lung injury, which is observed in hard metal lung disease [55,62]. Results of the effect of implantation of two different military grade W alloys in leg muscles of B6C3FI mice showed that W/Ni/Co alloy, but not W/Ni/Fe alloy induced the formation of a less aggressive and not metastasized rhabdomyosarcoma around the pellets. In contrast to the effects on rats, the formed tumor was not aggressive and did not metastasize [2]. Later, the same group used different singular or binary combinations of W, Co, and Ni to assess the role of each metal in the observed effects. Tantalum (Ta), an inert metal, was added to the single or bimetal alloy. Two years after implantation into the B6C3FI mice, W/Ta, Co/Ta and W/Ni/Ta alloy caused a lesser mortality rate compared to W/Co/Ni alloy. Also, sarcoma was formed in 20% of Co/Ta alloy-treated mice and 5% of W-Ta alloy-treated mice; however, sarcoma was present in 80% of W/Ni/Co-treated mice. These results showed a synergistic effect of the three metals in induction of the toxic effect [56]. An interesting observation of this study is that pellets containing W had a higher solubility rate compared to corresponding pellets that did not have W [56]. Also, the authors observed localization of these metals into brain and testes, indicating their ability to cross the blood-brain barrier and the blood–testes barrier [56]. In a follow-up study, tissue distribution analysis of W/Co/Ni implant in F344 rats after 6 months of exposure showed a systemic distribution, with the kidney, spleen, and liver having the highest metal concentrations. These results suggested that W alloy can contribute to chronic exposure to these metals [57] (Figure 3).

## 5. Conclusions

This review highlights the importance of taking the composition of a mixture and the chemical forms of its components into consideration while studying the safety of each component. Tungsten is used widely due to its unique characteristics including a higher melting point and a lower vapor pressure compared to other metals. Tungsten replaced lead in military applications such as the “green bullet”, but toxicological effects of tungsten are controversial. This confusion is enhanced considering the wide range of forms that have been tested in toxicological studies. Tungsten is usually used in the form of a mixture with other metals. Understanding the mechanisms of interaction of tungsten and those other metals will help minimize the adverse health effects of W-based products by changing the composition of the final products. Based on the current literature, the toxicity of WC-Co developed via solubilization of Co ions, interactions between W and Co surfaces, or internalization of the metal particles. Those proposed mechanisms have their own consequences. Although Co and Ni are important metals to provide the desirable characteristics of W-based alloy, the dissolution of Co and Ni ions from W/Ni/Co alloy is a driving cause of toxicity of this alloy. Dissolution of Ni and Co ions can induce production of ROS and DNA damage or induce HIF-1α stabilization and its downstream effects. As mentioned in this review, many factors can affect the potential health effects of tungsten compounds. Composition of the alloy, shape and size of particles, and even the ratio of the components could lead to different outcomes. It is important to note these factors during production and during toxicological assessment. Moreover, future investigations should consider the different exposure routes of the W/Ni/Co mixture. Most of the in vivo studies that are mentioned in this review only mimic the shrapnel wound effect, neglecting other routes of exposure such as contaminated drinking water.

## Figures and Tables

**Table 1:** Summary of the effects of tungsten compounds. Ap-1: activator protein; Co: cobalt; GSH: glutathione; HIF-1α: hypoxia inducible factor 1-α; MAPK: mitogen-activated protein kinase; Nf-KB: nuclear factor kappa-light-chain-enhancer of activated B; Ni: Nickel; NQO1: NADPH dehydrogenase quinone 1; Nrf2: nuclear factor erythroid 2-like factor 2; RBC: red blood cell; ROS: reactive oxygen species; W: Tungsten; WBC: white blood cell.

Compound/s	Model	Route of Exposure	Effects	Outcomes and Proposed mechanisms	Reference
Sodium tungstate	Immortalized human bronchial epithelial cells (Beas-2B)	- In vitro/cells grown in agar - Treated cells were implanted into flanks of mice	- Anchorage independent growth - Tumor formation - Upregulation of NQO1 and S-phase kinase associated protein - Inhibition of demethylases	Tumorgenicity Degradation of demethylases or induction of ROS	[11,15]
Tungstate-based shield	Breast cancer patients	Tungsten-based shield placed during intraoperative radiotherapy	Tungsten was detected in urine of patients with mastectomy	Mobilization of tungsten	[16]
Sodium tungstate	BALB/c mice injected with 66Cl4 cells	Tungsten added to drinking water	Lung metastasis	Activation of fibroblasts, myeloid derived suppressor cells, and matrix proteinases (changes in microenvironment of tumor)	[16]
Sodium tungstate	66Cl4 cells	In vitro	No change in proliferation	Tungsten does not directly induce tumor formation	[16]
Tungsten oxide microparticle and nanoparticle	Wistar rats	Oral administration	Induced DNA damage, micronuclei formation reduction of glutathione, and catalase	ROS, genotoxicity	[17]
Tungsten carbide nanoparticles	HepG2 and HaCat cells	In vitro	ROS and chromosomal instability	Small size, and presence of carbon black in WC particles	[49]
Tungsten carbide-cobalt	Human peripheral blood mononuclear cells	In vitro	Apoptosis, ROS, oxidative stress, inhibition of immune response	Induction of HIF-1α	[27,30,51]
Tungsten carbide-cobalt nanoparticles	HaCaT cells	In vitro	Increase transcription of genes involved in hypoxia pathways	Effects similar to Co ion’s effect	[41]
Tungstate carbide- cobalt nanoparticles	Mouse epidermal JB6 cells	In vitro	Activation Nrf2 and NQO1	ROS	[43]
Tungsten carbide-cobalt micro or nano particles	Beas-2B cells	In vitro	Decrease in cell viability, apoptosis, oxidative stress	Internalization of WC-Co inside the cells	[45]
Tungsten carbide-cobalt nanoparticles	JB6P + cells	In vitro	Activation of Ap-1, Nf-KB, MAPK, depletion of GSH	Oxidative stress	[47,48]
Tungsten alloy (W/Co/Ni)	F344 rats	Intramuscular implantation	Incidence of rhabdomyosarcoma, increase in RBCs, WBCs, neutrophils		[49]
W/Co/Ni or W/Co/Fe particles	L6-c11	In vitro	DNA damage, ROS	Dissolution of Co and Ni ions and stabilization of HIF-1α	[52]
W/Ni/Co or W/Ni/Fe pellets	F344 rats	Intramuscular implantation	Aggressive rhabdomyosarcomas formed around W/Co/Ni pellets only	Mobilization of the metal ions from the pellet	[53]
W/Ni/Co	hSKMC cells	In vitro	- Inhibition of muscle specific protein transcription - Induction of hypoxia, stress response, glycolysis, angiogenesis-related genes	Carcinogenesis	[54]
W/Ni/Fe or W/Ni/Co	Rat	Intratracheal instillation	ROS, induction of inflammatory cytokines	Phagocytosis of metal particles by lung macrophages	[55]
W/Ni/Co or W/Ni/Fe alloy	B6C3FI mouse	Implantation in leg muscles	Rhabdomyosarcoma formation around the W/Ni/Co pellet		[2]
W/Ta, Co/Ta, W/Ni/Co or W/Ni/Ta	B6C3FI mouse	Implantation in hind limb	- Formation of rhabdomyosarcoma tumors - Accumulation of the metals in brain, testes	- W/Ni/Co caused increased incidence of tumor compared to single or other mixtures - Ability of the metals to cross the blood–brain barrier and the blood–testis barrier	[56]
W/Ni/Co	F344 rats	Implantation	Systemic distribution of the metals. Liver, kidney and spleen are most affected organs		[57]
W, Co, Ni soluble single or paired metal salts	PCl2 cells	In vitro	Ni and Co in absence or presence of W led to changes in gene expression	W has minimal effect on the observed effects	[58]
W, Co, Ni	Hippocampal primary neuronal culture, mouse myoblast (C2C12)	In vitro	W/Ni/Co induced epigenetic alteration	W/Ni/Co synergistically caused the effect.	[59]
